# GPR68 Mediates Lung Endothelial Dysfunction Caused by Bacterial Inflammation and Tissue Acidification

**DOI:** 10.3390/cells13242125

**Published:** 2024-12-22

**Authors:** Pratap Karki, Yunbo Ke, Chenou Zhang, Kamoltip Promnares, Yue Li, Charles H. Williams, Charles C. Hong, Konstantin G. Birukov, Anna A. Birukova

**Affiliations:** 1 Division of Pulmonary and Critical Care, Department of Medicine, UMSOM Lung Biology Program, University of Maryland School of Medicine, 20 Penn Street, HSF-2, Room S143, Baltimore, MD 21201, USA; pkarki@som.umaryland.edu (P.K.); chen-ouzhange@som.umaryland.edu (C.Z.); yueli@som.umaryland.edu (Y.L.); 2 Department of Anesthesiology, University of Maryland School of Medicine, Baltimore, MD 21201, USA; yke@som.umaryland.edu (Y.K.); kpromnares@som.umaryland.edu (K.P.); kbirukov@som.umaryland.edu (K.G.B.); 3 Division of Cardiovascular Medicine, Department of Medicine, University of Maryland School of Medicine, Baltimore, MD 21201, USA; charles.williams@som.umaryland.edu (C.H.W.); charles.hong@som.umaryland.edu (C.C.H.)

**Keywords:** acidosis, endothelial permeability, inflammation, LPS, GPR68, OGM-8345, GPR4

## Abstract

Tissue acidification resulting from dysregulated cellular bioenergetics accompanies various inflammatory states. GPR68, along with other members of proton-sensing G protein-coupled receptors, responds to extracellular acidification and has been implicated in chronic inflammation-related diseases such as ischemia, cancer, and colitis. The present study examined the role of extracellular acidification on human pulmonary endothelial cell (EC) permeability and inflammatory status per se and investigated potential synergistic effects of acidosis on endothelial dysfunction caused by bacterial lipopolysaccharide (LPS, *Klebsiella pneumoniae*). Results showed that medium acidification to pH 6.5 caused a delayed increase in EC permeability illustrated by a decrease in transendothelial electrical resistance and loss of continuous VE-cadherin immunostaining at cell junctions. Likewise, acidic pH induced endothelial inflammation reflected by increased mRNA and protein expression of EC adhesion molecules VCAM-1 and ICAM-1, upregulated mRNA transcripts of tumor necrosis factor-α, IL-6, IL-8, IL-1β, and CXCL5, and increased secretion of ICAM-1, IL-6, and IL-8 in culture medium monitored by ELISA. Among the GPCRs tested, acidic pH selectively increased mRNA and protein expression of GPR68, and only the GPR68-specific small molecule inhibitor OGM-8345 rescued acidosis-induced endothelial permeability and inflammation. Furthermore, acidic pH exacerbated LPS-induced endothelial permeability and inflammatory response in cultured lung macrovascular as well as microvascular endothelial cells. These effects were suppressed by OGM-8345 in both EC types. Altogether, these results suggest that GPR68 is a critical mediator of acidic pH-induced dysfunction of human pulmonary vascular endothelial cells and mediates the augmenting effect of tissue acidification on LPS-induced endothelial cell injury.

## 1. Introduction

Ischemic and inflammatory conditions stimulate lactate production via anaerobic glycolysis that leads to extracellular acidification [1]. The co-existence of tissue acidification and inflammation during various pathological conditions suggests that extracellular pH may be an active and direct modulator of immune function [2]. Besides proton-sensing ion channels, a family of G protein-coupled receptors (GCPRs) comprising GPR4, GPR65, or T-cell death-associated gene 8 (TDAG8), GPR68, or ovarian cancer GPCR1 (OGR1), and GPR132 also function as proton sensors [3,4]. Among these GPCRs, GPR4 and GPR68 show more widespread expression across various organs, including vascular endothelial cells (ECs). Indeed, a few studies have already demonstrated that acidosis-induced activation of GPR4 mediates increased endothelial permeability, inflammatory response, and endoplasmic reticulum stress in ECs [5,6,7,8]. A recent study has suggested the involvement of GPR68 expressed in microvascular ECs in controlling flow-mediated EC-dependent dilation of mesenteric arteries in mice [9]. However, a precise role of GPR68 in mediating acidosis or inflammatory agonist-induced endothelial dysfunction remains unknown.

Severe lung inflammation and increased lung endothelial permeability are major features of acute respiratory distress syndrome (ARDS), a life-threatening respiratory illness developing in various pathological settings, including sepsis, pneumonia, massive trauma, toxic lung injury, and conditions [10,11]. The high mortality rate of ~40% and recent observations that ARDS contributes to the severity of the current global pandemic COVID-19 highlight the urgency of drug development for this devastating condition [12,13,14]. In this regard, proton-sensing GPCRs may be considered as potential therapeutic targets in ARDS, given that acidosis triggers inflammation [3,15]. This notion is further supported by the findings that GPR68 is involved in the pathogenesis of intestinal inflammation, fibrosis, and colitis, indicating its potential, yet unexplored role in the propagation of lung inflammation [16,17].

In this study, we analyzed the role of GPR68 in mediating acidosis-induced permeability and inflammation in primary cultures of human lung ECs and evaluated the synergistic effects of acidification and bacterial lipopolysaccharide (LPS) in exacerbating endothelial dysfunction. Furthermore, we evaluated the protective effects of a novel first-in-class GPR68 ogremorphin inhibitor, OGM-8345, against acidosis- and LPS-induced endothelial permeability and inflammation.

## 2. Materials and Methods

### 2.1. Reagents

LPS (*Klebsiella pneumoniae*, ATCC 15380) and Ogerin were purchased from Sigma (St. Louis, MO, USA). GPR4 inhibitor NE52-QQ57 was from MedKoo Biosciences Inc. (Morrisville, NC, USA), and GPR68 inhibitor OGM-8345 was routinely synthesized in our lab. and dissolved in DMSO (10–40 mg/mL) as described recently [18]. Antibodies to VE-cadherin and ICAM-1 were obtained from Santa Cruz Biotechnology (Santa Cruz, CA, USA). Antibodies to IkBα, phospho- and total NF-kB, VCAM-1, as well as HRP-linked anti-mouse and anti-rabbit secondary antibodies were from Cell Signaling (Beverly, MA, USA). GPR68 antibody was obtained from Thermo Fisher Scientific (Waltham, MA, USA).

### 2.2. Cell Culture and Acidosis Stimulation

Primary cultures of human pulmonary artery endothelial cells (HPAECs), human lung microvascular endothelial cells (HLMVEC), and EGM-2 growth media kit were obtained from Lonza (Allendale, NJ, USA). Cells were used at passages 5–8, and experimental stimulations were performed in EGM-2 containing 2% fetal bovine serum. For acidosis stimulation, the pH of 20 mM HEPES-buffered culture medium was adjusted to desired values with HCl or NaOH using an electronic pH meter. Medium was equilibrated in a cell culture incubator (37 °C, 5% CO_2_) for 2–3 h prior to use.

### 2.3. Endothelial Permeability Measurements

Electric cell–substrate impedance sensing system (ECIS, Applied Biophysics, Troy, NY, USA) was employed to measure transendothelial electrical resistance (TER) across EC monolayers as described previously [19]. On the day of the TEER experiment, the culture medium in the EC monolayer grown on ECIS plates was replaced with EGM-2 containing 2% serum, and TEER equilibration was reached within 30–45 min. At this point, TEER recording restarted, and after 10–15 min, desired stimulations were performed. TEER measurements were continued for an additional 16–25 h. Only cells reaching >1200 ohms of steady-state resistance were used for TER analysis, and normalized resistance was plotted against time to determine EC permeability. For each condition, 3–6 independent experiments were performed.

Similarly, EC permeability to macromolecules was determined by express permeability testing (XPerT) assay developed by our group [20]. Briefly, FITC-avidin solution was added directly to the culture medium for 3 min before termination of the experiment, and unbound FITC-avidin was washed out with PBS (pH 7.4, 37 °C). Cells were fixed with 3.7% formaldehyde in PBS (10 min, room temperature), and images of FITC-avidin bound to the biotinylated gelatin matrix were captured using a Nikon Eclipse TE 300 microscope (Nikon, Tokyo, Japan).

### 2.4. Immunocytochemistry

Immunostaining with VE-cadherin antibody was performed to visualize EC junctions. In brief, cells were fixed in 3.7% formaldehyde solution for 10 min. at 4 °C, followed by permeabilization with 0.1% Triton X-100 for 30 min at room temperature. Blocking was performed with 2% BSA in PBS for 30 min followed by incubation with VE-cadherin antibody for 1 h at room temperature. Secondary antibody staining was performed with appropriate Alexa 488-conjugated antibody, and Texas Red-conjugated phalloidin was also added to visualize actin filaments. Slides were analyzed using a Nikon Eclipse TE300 inverted microscope connected to an SPOT RT monochrome digital camera and image processor (Diagnostic Instruments, Sterling Heights, MI, USA). Images were processed using Adobe Photoshop 7.0 (Adobe Systems, San Jose, CA, USA).

### 2.5. GPR68 Activation Assay

The PRESTO-Tango assay was run to determine GPR68 activation as described earlier [21]. Briefly, cells were seeded on 6-well dishes, and transfection was carried out with a combination of GPR68 Tango, β-arrestin, and luciferase plasmids using LTX Lipofectamine reagent (Thermo Fisher Scientific) for 24 h. At the end of desired periods of agonist stimulation, a luciferase assay was performed with Bright-Glo reagent from Promega by measuring luminescence on a VICTOR X5 multiplate reader (PerkinElmer, Waltham, MA, USA).

### 2.6. Western Blotting

Cells were washed with PBS and lysed directly in Laemmli sample buffer. Cell lysates were run on 8% SDS-PAGE and transferred onto polyvinylidene fluoride membranes using a semi-dry transfer system (17 V, 1 h, Bio-Rad, Hercules, CA, USA). Blocking of membranes was performed in 3% BSA for 1 h at room temperature and incubated with desired primary antibodies at 4 °C overnight. Horseradish peroxidase-conjugated secondary antibodies were added at room temperature for 1 h. Immunoreactive protein bands were detected using an enhanced chemiluminescent system (Thermo Fisher Scientific). Image J software (Version 1.54, NIH, Bethesda, MD, USA) was utilized to calculate image intensities.

### 2.7. Quantitative Real-Time PCR

RNeasy Plus Kit (Qiagen, Germantown, MD, USA) was used to isolate total RNA, and one microgram of cDNA was synthesized with the iScript cDNA synthesis kit (Bio-Rad, Hercules, CA, USA). Then, quantitative real-time PCR was performed on a Bio-Rad CFX96 real-time PCR system with SYBR green (Quantabio, Beverly, MA, USA). Ct values were normalized to GAPDH, and fold changes in gene expression were calculated using the ΔΔCt method. Primers used in qPCR are listed in Table 1.

### 2.8. ELISA

ELISA was performed to determine the secretory levels of sICAM, IL-6, and IL-8 in conditioned media with commercially available kits (R&D Systems, Minneapolis, MN, USA). Briefly, supernatants obtained after brief centrifugation (800× *g*, 3 min) were run for ELISA following the manufacturer’s instructions. Absorbance was read at 450 nm within 30 min., and the concentration of cytokines was calculated by generating a standard curve.

### 2.9. Statistical Analysis

Results are expressed as mean ± SD or SE of three to eight independent experiments. Continuous variables were compared using Student’s *t*-test or Mann–Whitney U test depending on normality. For multiple-group comparisons, one-way ANOVA or the Kruskal–Wallis test was used. In all tests, *p* < 0.05 was considered statistically significant.

Control and stimulated groups were compared using an unpaired Student’s *t*-test. For multiple group comparisons, one-way ANOVA followed by the post hoc Fisher’s test was used with *p* < 0.05 considered as statistically significant.

## 3. Results

### 3.1. Acidosis Induces Endothelial Dysfunction

**Figure 1:**
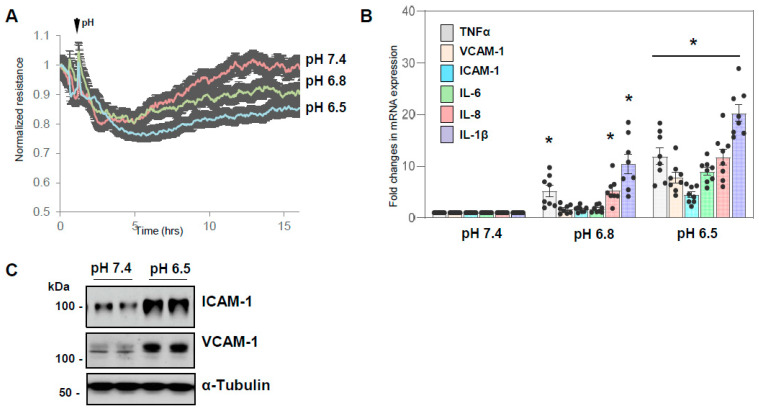
Acidosis induces endothelial dysfunction. (**A**) TEER measurements in HPAEC monolayers incubated at normal pH 7.4 (red) or lowered pH levels: pH 6.8 (green) and pH 6.5 (blue). Time point of pH change is marked by arrow. TEER changes were monitored over time; *n* = 4. (**B**) qRT-PCR analysis of mRNA expression of inflammatory marker genes: TNF-α, VCAM-1, ICAM-1, IL-6, IL-8, and IL-1β in HPAECs cultured in media with normal and acidic pH for 3 h. The histograms represent single points, mean (bar), and SD values; * *p* < 0.05, vs. pH 7.4; *n* = 6–8. (**C**) Western blot analysis of ICAM-1 and VCAM-1 protein expression by HPAECs incubated for 6 h under control (pH 7.4) or acidic (pH 6.5) conditions; probing for α-tubulin was used as a loading control. Shown are representative data of 5 independent experiments.

Initial experiments were designed to evaluate the effects of medium acidification on endothelial function. Measurements of TER showed that there was a gradual increase in HPAEC permeability with pH shift to acidic range (Figure 1A). Consistently, lowering the cell medium pH from normal physiological level (pH 7.4) to pH 6.8 led to an increase in mRNA levels of endothelial inflammatory marker genes: TNF-α, VCAM-1, ICAM-1, IL-6, IL-8, and IL-1β, which was further enhanced at pH 6.5 (Figure 1B). In agreement with qRT-PCR data, Western blot analysis showed increased protein expression of ICAM-1 and VCAM-1 (Figure 1C). Altogether, these data suggest that acidosis may trigger increased EC permeability and inflammatory response.

### 3.2. GPR68 Mediates Acidosis-Induced Endothelial Dysfunction

**Figure 2:**
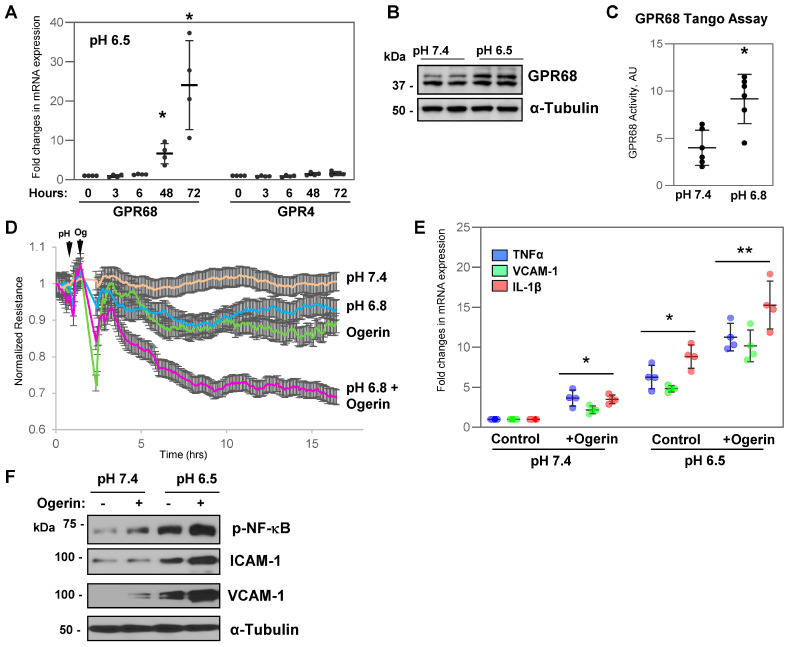
Acidosis activates GPR68, leading to endothelial dysfunction. (**A**) HPAECs were switched to acidic pH medium for indicated time periods. mRNA transcript levels of GPR68 and GPR4 were analyzed by qRT-PCR. The histograms represent single points, mean, and SD values; * *p* < 0.05, vs. pH 7.4; *n* = 4. (**B**) Western blot analysis of GPR68 protein levels of HPAECs grown in normal or acidic media for 24 h; probing for α-tubulin was used as a loading control. (**C**) HPAECs cultured at the indicated pH were transfected with a combination of GPR68 PRESTO-Tango plasmids for 24 h, followed by monitoring GPR68 activity by luciferase assay; * *p* < 0.05, vs. pH 7.4 control. (**D**) HPAECs were exposed to acidic pH (first arrow) and were stimulated with Ogerin (10 µM, second arrow), and endothelial permeability was monitored by TER changes over time; *n* = 3. (**E**) HPAECs incubated at normal or acidic pH were treated with Ogerin (10 µM, 3 h), and mRNA levels of TNF-α (blue), VCAM-1 (green), and IL-1β (red) were determined by qRT-PCR. * *p* < 0.05, vs. pH 7.4 control, ** *p* < 0.05, vs. Ogerin at pH 7.4; *n* = 4. (**F**) HPAECs were stimulated with Ogerin (10 µM, 6 h) at pH 7.4 or pH 6.5 followed by Western blot analysis of phospho-NFkB, ICAM-1, and VCAM-1 protein expression levels; probing for α-tubulin was used as a loading control. Shown are blots representative of three independent experiments.

Prior studies have suggested a role of GPR68 in intestinal inflammation [16,17], but its role in lung endothelial dysfunction has not been yet investigated. Analysis of GPR68 expression in HPAECs exposed to acidified medium (pH 6.5) showed a time-dependent increase in mRNA expression of GPR68 (Figure 2A). In contrast, exposure to acidic pH did not affect the mRNA levels of GPR4. Protein expression analysis further confirmed enhanced expression of GPR68 when culture medium was switched from pH 7.4 to pH 6.5 (Figure 2B). The assessment of GPR68 receptor activation using the PRESTO-Tango assay also demonstrated that lowering pH favors GPR68 activation (Figure 2C). To further validate the role of GPR68 in acidosis-induced endothelial inflammation, we used Ogerin, a pharmacological compound that selectively activates GPR68 [22]. TER measurements revealed that lowering media pH to 6.8 or exposure to Ogerin increased HPAEC permeability, and the effect was robustly escalated when both stimuli were combined (Figure 2D). Likewise, Ogerin increased mRNA expression of pro-inflammatory molecules TNF-α, VCAM-1, and IL-1β, albeit slightly lower than acidic pH alone (Figure 2E). The combination of Ogerin and acidic pH caused an additional increase in transcriptional activation of inflammatory markers. Consistently, Ogerin-induced NF-kB phosphorylation and increased expression of ICAM-1 and VCAM-1 proteins were markedly increased at pH 6.5 from that of pH 7.4 (Figure 2F). Similar results were observed in HLMVEC isolated from the lung microvascular bed. Collectively, these findings strongly indicate that acidosis activates pH sensor GPR68 in EC, which mediates acidosis-induced endothelial dysfunction.

### 3.3. OGM-8345 Rescues Acidosis-Induced Endothelial Dysfunction

**Figure 3:**
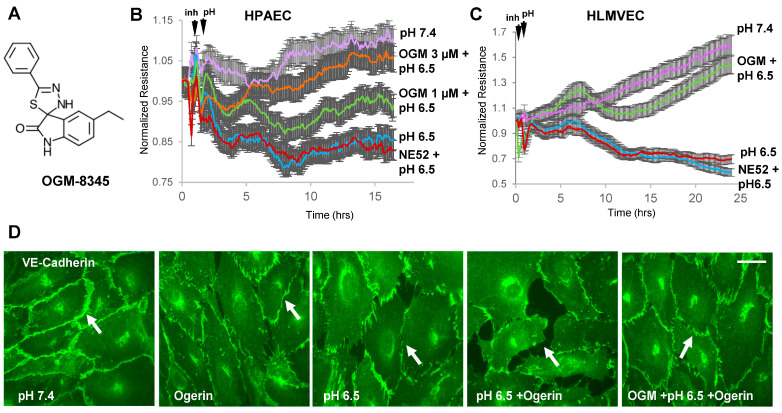
Inhibition of GPR68 but not GPR4 rescues acidosis-induced endothelial barrier disruption. (**A**) Chemical structure of GPR68 inhibitor OGM-8345. (**B**) HPAECs were switched to acidic pH media and treated with OGM-8345 (1 µM and 3 µM) or NE52-QQ57 (1 µM). TER was monitored over time, and normalized resistance is presented; *n* = 4. (**C**) Human lung microvascular endothelial cells (HLMVEC) were exposed to acidic pH medium and treated with OGM-8345 (3 µM) or NE52-QQ57 (1 µM). Endothelial permeability was determined by measuring TER over time; *n* = 5. (**D**) HPAECs were exposed to acidic pH or Ogerin alone or to their combination, with or without OGM-8345 (3 µM, 6 h). Immunostaining for VE-cadherin was performed to visualize endothelial adherens junctions. Bar: 10 µm. Changes in adherence junctions’ architecture are shown by arrows.

As our results suggested that GPR68 activation enhances acidosis-induced endothelial barrier disruption and inflammation, we next tested the hypothesis that GPR68 inhibition rescues acidic pH-derived endothelial dysfunction. For this purpose, we employed OGM-8345 (Figure 3A), a GPR68-specific small molecule inhibitor recently developed by our group [18,23]. OGM-8345 caused pronounced and dose-dependent attenuation of HPAEC permeability caused by acidic pH, while the GPR4 inhibitor NE52-QQ57 was without effect (Figure 3B). A strong protective effect of OGM-8345 against acidic pH-induced EC barrier disruption was also observed in lung microvascular EC (Figure 3C), while GPR4 inhibitor failed to prevent permeability increase. OGM-8345-mediated EC barrier protection was further evaluated by analysis of EC adherens junction integrity using immunostaining of adherens junction protein VE-cadherin. Ogerin or acidic pH alone disrupted the continuous pattern of VE-cadherin staining with the appearance of paracellular gaps (Figure 3D). Breakdown of EC junctions was further augmented when both stimuli were combined. Importantly, EC pre-treatment with OGM-8345 attenuated the disruption of EC junctions caused by acidic pH and Ogerin, reflected by the preservation of a continuous VE-cadherin staining pattern and prevention of paracellular gap formation (Figure 3D).

**Figure 4:**
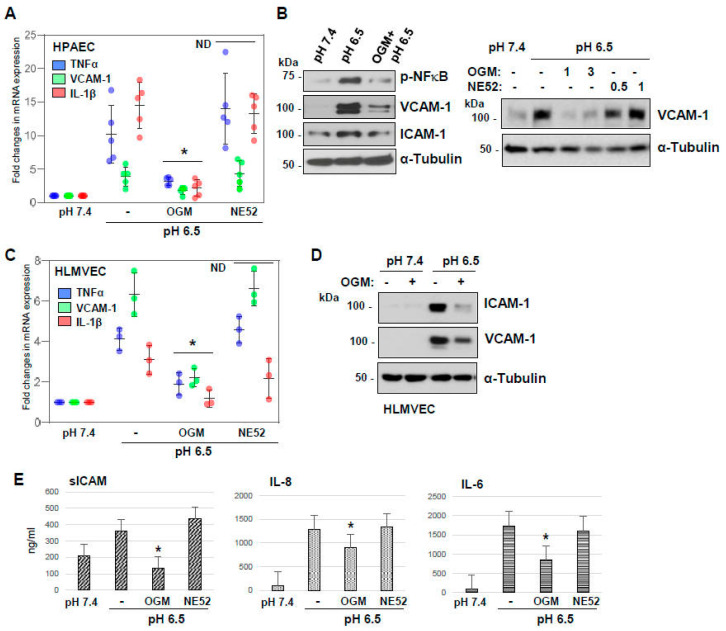
OGM-8345 but not NE52 attenuates acidosis-induced endothelial inflammatory responses. (**A**) HPAECs were switched to acidic pH medium alone or supplemented with indicated GPCRs inhibitors. After 3 h of incubation, mRNA expression analysis of TNF-α, VCAM-1, and IL-1β was performed by qRT-PCR. * *p* < 0.05, vs. pH 6.5, ND: statistically not significant compared to pH 6.5; *n* = 5. (**B**) HPAECs were incubated in acidic pH media with or without OGM-8345 (3 µM, 6 h); the levels of NFkB phosphorylation and VCAM-1 and ICAM-1 protein expression were determined by Western blot; α-tubulin was used as a loading control (**left panel**). VCAM-1 protein levels were determined in cells incubated at acidic pH with indicated doses (in µM) of OGM-8345 or NE52-QQ57 (**right panel**). (**C**,**D**) HLMVEC were incubated in acidic pH medium alone or with OGM-8345 or NE52-QQ57 where indicated. (**C**) After 3 h, mRNA levels of TNF-α, VCAM-1, ICAM-1, and IL-1β were determined by qRT-PCR * *p* < 0.05, vs. pH 6.5, ND: statistically not significant compared to pH 6.5; *n* = 3. (**D**) Western blot analysis of ICAM-1 and VCAM-1 protein expression; reprobing with α-tubulin served as a loading control. (**E**) HPAECs exposed to acidic pH media with or without indicated inhibitors for 6 h were subjected to ELISA analysis to determine the secretory levels of sICAM, IL-8, and IL-6; * *p* < 0.05, vs. pH 6.5, *n* = 3.

In the next sets of experiments, we investigated whether OGM-8345 possesses anti-inflammatory activities and rescues acidic pH-stimulated endothelial inflammation. The results showed that transcriptional activation of TNF-α, VCAM-1, and IL-1β stimulated by pH 6.5 was blocked by treatment with OGM-8345 (Figure 4A). However, the GPR4 inhibitor NE52-QQ57 did not affect the HPAECs’ inflammatory response to medium acidification. Similarly, acidosis-induced activation of inflammatory signaling reflected by increased NFκB phosphorylation and increased protein expression of VCAM-1 and ICAM-1 was attenuated by OGM-8345 (Figure 4B). The contrasting effects of GPR68 and GPR4 receptor inhibitors were further verified in experiments with acidic pH-induced VCAM-1 protein expression, which confirmed the inefficiency of GPR4 inhibition to prevent pH-induced inflammatory activation in HPAECs (Figure 4B, right panel). Inflammatory response to medium acidification was not limited to ECs from larger blood vessels, as acidic pH also enhanced the mRNA transcript levels of inflammatory genes TNF-α, VCAM-1, ICAM-1, and IL-1β in human lung microvascular EC (Figure 4C). This effect observed in HLMVEC was strongly inhibited by OGM-8345 but not by NE52-QQ57. Western blot analysis of ICAM1 and VCAM1 protein expression further confirmed the efficacy of OGM-8345 but not NE52-QQ57 in inhibiting acidic pH-induced upregulation of ICAM-1 and VCAM-1 proteins in microvascular EC (Figure 4D). The anti-inflammatory effect of OGM-8345 was further illustrated by significant inhibition of acidic-pH-induced secretion of soluble ICAM, IL-6, and IL-8 by pulmonary EC, which was detected by ELISA assays of conditioned medium (Figure 4E). Yet, NE52-QQ57 failed to inhibit acidification-induced cytokine secretion.

### 3.4. Acidosis Exacerbates LPS-Induced Endothelial Permeability: Role of GPR68

**Figure 5:**
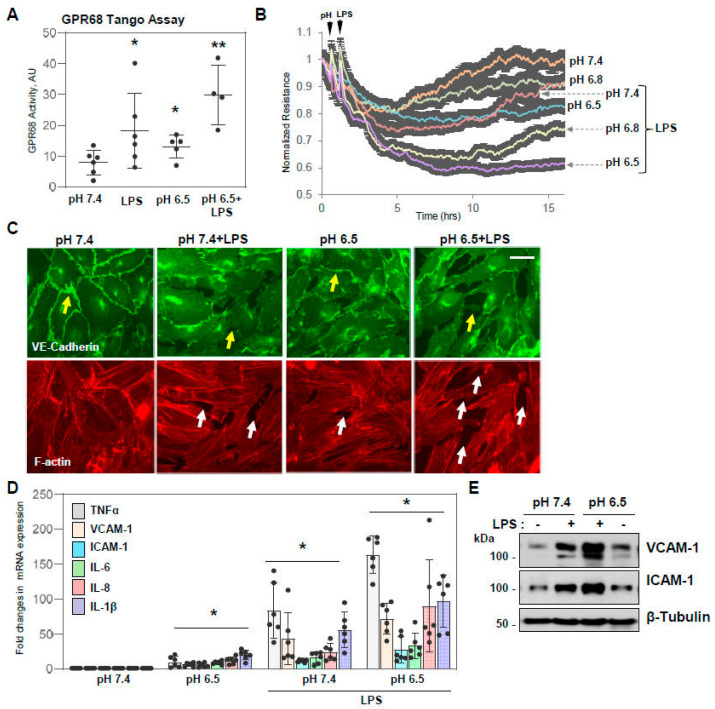
LPS exacerbates acidosis-induced endothelial dysfunction. (**A**) HPAECs incubated at normal or acidic pH were stimulated with LPS (100 ng/mL, 6 h) followed by measurement of GPR68 activity by Tango assay. * *p* < 0.05, vs. pH 7.4 control, ** *p* < 0.05, vs. pH 7.4+LPS, *n* = 4–6. (**B**) Cells incubated in the indicated pH media were stimulated with vehicle or LPS, and TER was monitored over time; *n* = 4. (**C**) HPAECs were stimulated with LPS (100 ng/mL, 6 h) in normal (pH 7.4) or acidic (pH 6.5) medium. Immunofluorescence staining with VE-cadherin antibody was performed to visualize adherens junctions. Changes in adherence junctions’ architecture are shown by yellow arrows; staining for F-actin was used to monitor actin cytoskeleton remodeling and paracellular gap formation (shown by white arrows). Bar: 10 µm. (**D**,**E**) HPAECs exposed to normal or acidic pH were treated with LPS (50 ng/mL) followed by qRT-PCR analysis of mRNA expression of indicated pro-inflammatory marker genes. (**D**); and by Western blot analysis of VCAM-1 and ICAM-1 protein levels (**E**). * *p* < 0.05, vs. pH 7.4 control, ** *p* < 0.05, vs. pH 7.4+LPS, *n* = 5–6.

Because extracellular acidification is typically associated with the onset of inflammatory response to infection [24], we examined if these two factors act in synergy to cause endothelial dysfunction. GPR68 activation was measured by the PRESTO-Tango assay under normal (pH 7.4) and acidic (pH 6.5) pH levels with or without adding LPS. The results showed that LPS-induced GPR68 activation was further augmented by pH acidification (Figure 5A). The measurements of TER in HPAEC monolayers showed that LPS-induced barrier disruption was gradually augmented by changing the pH of culture medium from 7.4 to 6.8 and 6.5 (Figure 5B). LPS and acidic pH also acted synergistically in causing the breakdown of EC adherens junctions and paracellular gap formation, as visualized by VE-cadherin and F-actin immunofluorescence staining (Figure 5C). Similarly, lowering pH to 6.6 augmented the HPAEC inflammatory response to LPS, reflected by increased mRNA expression of TNF-α, VCAM-1, and ICAM-1 and protein expression of VCAM-1 and ICAM-1 (Figure 5D,E).

**Figure 6:**
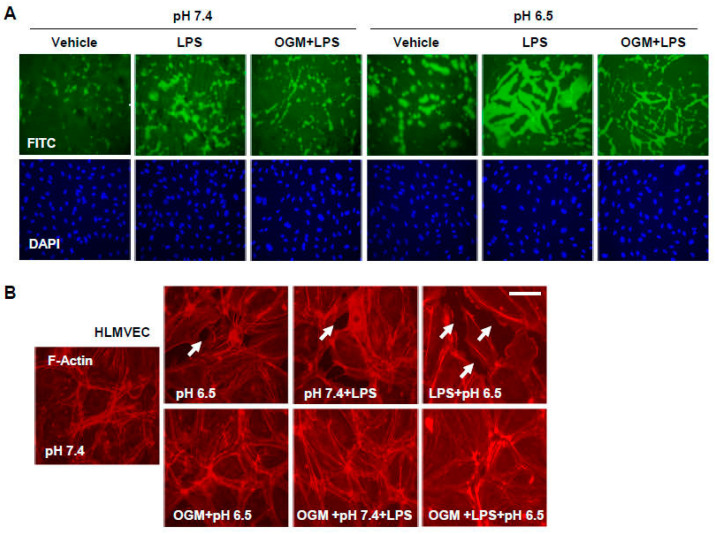
OGM-8345 attenuates LPS-induced endothelial barrier disruption at normal and acidic pH. (**A**) HPAECs grown in normal or acidic pH media were pre-treated with OGM-8345 (1 µM) followed by the addition of LPS (100 ng/mL) for 6 h. XPerT assay was performed to determine endothelial macromolecular permeability. FITC fluorescence micrographs are presented; DAPI-counterstaining depicts cell nuclei. (**B**) HLMVEC were stimulated with LPS (100 ng/mL, 6 h) in normal or acidic media in the presence or absence of OGM-8345 (1 µM). F-actin staining was performed to monitor cytoskeletal remodeling and paracellular gap formation (shown by arrows). Shown are representative results of 4 independent experiments; bar: 10 µm.

Alternative analysis of endothelial barrier dysfunction by the XPerT assay demonstrated the synergistic effect of LPS and culture medium acidification from pH 7.4 to pH 6.5 on the permeation of FITC-labeled tracer through the EC monolayer, reflecting a rise in macromolecular permeability (Figure 6A). This permeability increase was strongly attenuated by OGM-8345, illustrated by a sharp decrease in green fluorescence signal at intercellular areas (Figure 6A).

A variability across vascular beds of different organs and between EC sub-phenotypes is well recognized [25,26]. In order to test the broader specificity of OGM-8345 in rescuing acidosis-induced EC cytoskeletal remodeling and barrier dysfunction, we examined its barrier protective effects in lung microvascular EC. F-actin staining verified that LPS-induced actin stress fiber formation and appearance of paracellular gaps were more expressed at acidic pH as compared to normal pH, but OGM-8345 was equally effective in attenuating these cytoskeletal changes in microvascular EC (Figure 6B).

### 3.5. GPR68 but Not GPR4 Mediates Synergistic Effects of Acidosis and LPS on Endothelial Inflammation

**Figure 7:**
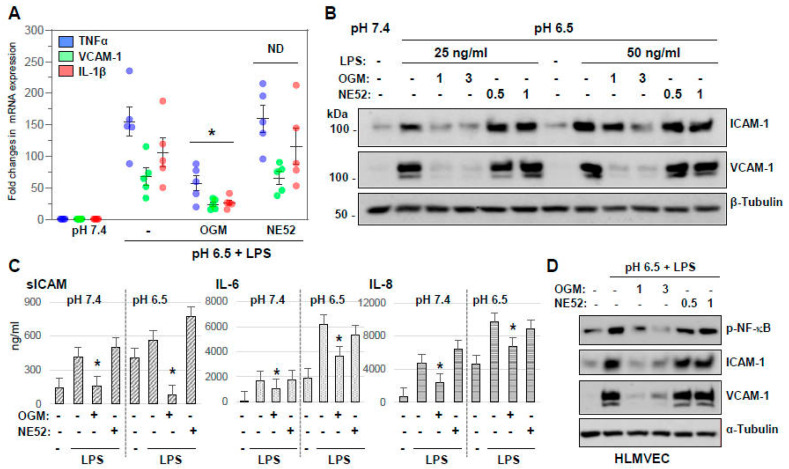
OGM-8345, but not NE52-QQ57, attenuates acidosis- and LPS-induced endothelial inflammation. (**A**) HPAECs pre-treated with OGM-8345 or NE52 (30 min) were switched to media with pH 6.5 followed by stimulation with LPS (50 ng/mL, 3 h). qRT-PCR analysis was carried out to determine mRNA transcript levels of TNF-α (blue), VCAM-1 (green), and IL-1β (red). * *p* < 0.05, vs. pH 6.5+LSP, ND—statistically not significant; *n* = 5. (**B**) HPAECs were pre-incubated with OGM-8345 (1 or 3 µM) or NE52-QQ57 (0.5 or 1 µM) at acidic pH followed by the addition of LPS (50 ng/mL, 6 h). Protein expression of ICAM-1 and VCAM-1 was determined by Western blot; β-tubulin was used as a loading control. (**C**) HPAECs pre-treated with OGM-8345 or NE52 (1 µM, 30 min) were incubated at normal or acidic pH followed by the addition of LPS (50 ng/mL, 6 h). Secretory protein levels of sICAM, IL-6, and IL-8 in conditioned media were evaluated by ELISA assay. * *p* < 0.05, vs. corresponding LPS groups, *n* = 3. (**D**) HLMVEC were pre-incubated with indicated doses (in µM) of GPR68 and GPR4 inhibitors at pH 6.5 media for 30 min followed by LPS stimulation (100 ng/mL, 6 h). Cell lysates were subjected to Western blot analysis to determine protein levels of phospho-NFkB, ICAM-1, and VCAM-1; reprobing for α-tubulin was used as a loading control. Shown are representative results of 4 independent experiments.

The impact of GPR68 and GPR4 receptors on LPS- and acidosis-induced EC inflammatory response was further evaluated by using selective GPR68 and GPR4 inhibitors. qRT-PCR analysis showed that EC combined exposure to pH 6.5 and LPS strongly increased mRNA levels of TNF-α, VCAM-1, ICAM-1, IL-6, IL-8, and IL-1β; this effect was only attenuated by EC pre-treatment with OGM-8345, but not with NE52-QQ57 (Figure 7A). Likewise, an increase in protein levels of VCAM-1 and ICAM-1 caused by acidic pH 6.5 and LPS was inhibited by two different doses of OGM-8345 without any effect exhibited by NE52-QQ57 (Figure 7B). Measurement of pro-inflammatory cytokines in EC-conditioned media showed that LPS-induced secretion of soluble ICAM (sICAM), IL-6, and IL-8 was more pronounced at acidic pH 6.5 compared to normal physiologic pH 7.4 (Figure 7C). OGM-8345 significantly attenuated LPS-induced increases in sICAM, IL-6, and IL-8 secretion at both physiologic and acidic pH, but GPR4 inhibitor had no effect on all conditions (Figure 7C). The selective inhibitory effect of OGM-8345 was further validated in lung microvascular EC. Similarly to HPAECs, LPS-induced expression of phospho-NF-κB, VCAM-1, and ICAM-1 proteins by HLMVEC was further augmented at acidic pH and was efficiently blocked by two different doses of OGM-8345, while NE52-QQ57 failed to interfere with these inflammatory responses (Figure 7D). Collectively, these data provide compelling evidence that inhibition of GPR68 but not GPR4 rescues acidosis-induced dysfunction of human pulmonary macrovascular and microvascular EC.

## 4. Discussion

Emerging evidence suggests an essential role of proton-sensing GPCRs in vascular pathophysiology, although their direct involvement in endothelial function remained unclear. Herein, we present the evidence that GPR68 is not only the critical mediator of acidosis-induced lung endothelial dysfunction but also drives synergistic pro-inflammatory effects of acidic pH and LPS leading to severe endothelial dysfunction. This notion is supported by the following findings: (i) acidic pH 6.5 increased permeability and triggered an inflammatory response by pulmonary EC; these effects were rescued by the small molecule inhibitor of GPR68, OGM-8345, but not by the GPR4 inhibitor NE52-QQ57; (ii) acidosis specifically upregulated transcriptional activation and protein expression of GPR68, while Ogerin, a synthetic GPR68 activator, further augmented endothelial dysfunction caused by medium acidification; and (iii) LPS stimulation of EC exacerbated acidic pH-induced endothelial damage; this effect was attenuated selectively by the GPR68 inhibitor.

A complex interplay exists between inflammation and related tissue acidosis, as excessive inflammatory responses contribute to the development of an acidic microenvironment, and acidification promotes inflammation [27]. Studies have shown an enhancement of inflammatory responses along with the release of pro-inflammatory cytokines in response to acidic pH, especially by immune cells [28,29]. Endothelial inflammation is a major pathological hallmark of ARDS, sepsis, and the pathogenesis of COVID-19 [30], but the impact of acidic pH on lung EC function remains largely unexplored. Interestingly, proton-sensing GPCRs have been suggested to mediate vascular, cardiac, and intestinal inflammation in various experimental settings [16,17,31,32,33,34]. In this context, we performed a comprehensive analysis of the effects of lowered pH on lung endothelial permeability and inflammation as well as the co-operative effects of pH and ARDS-related inflammatory agonist on endothelial dysfunction. We also evaluated the effectiveness of our newly developed GPR68 small molecular inhibitor OGM-8345 in rescuing acidosis-driven endothelial dysfunction.

Our results show that acidosis alone is sufficient to cause endothelial damage as evidenced by a gradual increase in endothelial permeability with pH shift from 7.4 to 6.5 and a similar trend in augmenting the EC inflammatory response. On analyzing the involvement of various proton-sensing GPCRs, we found that acidic pH selectively activates GPR68, demonstrating several-fold increases in its mRNA as well as protein levels. Notably, GPR4, which is also abundantly expressed in EC and was earlier reported to mediate vascular endothelial dysfunction [5,7], did not respond to reduction in pH in both macrovascular and microvascular lung ECs in our experimental settings. The reason behind these discrepancies is not clear at this moment, and whether different experimental approaches solely contributed to these inconsistencies or other factors, such as EC heterogeneity and their variable responses to agonists [25,35], were also reasons behind these outcomes is still unknown. In support of our notion, an essential role of GPR68 in acidosis-triggered endothelial dysfunction was further verified by the observations that Ogerin, a synthetic GPR68 activator, augmented acidic pH-induced endothelial inflammation. Moreover, assessment of pharmacological inhibitors of GPR68 and GPR4 on acidic pH-caused endothelial dysfunction further assisted to delineate the involvement of these receptors. Our small molecule GPR68-specific inhibitor OGM-8345 potently blocked acidic pH-induced endothelial barrier disruption as well as inflammatory responses in lung EC (Figure 4). In sharp contrast, the commercially available GPR4 inhibitor NE52-QQ57 failed to show any protective effects against pH-induced endothelial damage. These findings were verified by multiple complementary assays of endothelial function measurements, such as TER monitoring, XPerT assay, mRNA/protein expression analysis of inflammatory marker genes, and determination of secretory levels of pro-inflammatory cytokines by ELISA. Furthermore, consistent observations of strong protective effects of OGM-8345 but no such beneficial activity of GPR4 inhibitor against acidic pH-induced endothelial damage in lung microvascular EC supported our statement that GPR68 is the major mediator of acidosis-induced endothelial dysfunction, at least in our experimental settings. In line with our findings, an earlier study has reported the activation of GPR68 during hypoxia-mediated acidic conditions in immune cells [36]. The mechanisms of acidosis-induced and GPR68-mediated endothelial dysfunction await further elaborated studies, but our data are in line with the classical pathways of agonist-induced endothelial barrier breach via breakdown of EC junctions and cytoskeletal remodeling with paracellular gap formations [37,38]. A wide array of pro-inflammatory cytokines was upregulated in response to acidic pH in EC, pointing towards the involvement of multiple inflammatory signaling cascades. Regarding the role of acidosis in inducing inflammatory pathways, an earlier study had demonstrated that extracellular acidification activates NLRP3 inflammasome leading to the secretion of IL-1β and caspase-1 activation in immune cells [39].

We also investigated the exacerbating effects of acidosis on inflammatory agonist-induced endothelial dysfunction since acidosis and inflammation are inter-related events. Our data exhibited strong synergistic effects of acidic pH and LPS on causing endothelial hyperpermeability and inflammatory responses. These findings may reflect an important clinical scenario where the inflammation-generated acidic environment of tissues further deteriorates the health of an individual, especially in critically ill patients [27,40]. Conversely, excessive acid generated from dysregulated metabolic states can lead to induction or exacerbation of existing inflammatory responses. Thus, a vicious cycle of acidosis and inflammation may occur in the lungs, being the primary organ for gaseous exchange regulating acid–base balance and also a hub for immune cells to tackle bacterial pathogens or other stimuli-initiated inflammatory cascades. Our results also showed that acidic pH augments LPS-induced mRNA expression of GPR68 but has no effect on GPR4 expression (Figure 6). Again, inhibition of GPR68 with OGM-8345 successfully attenuated LPS-induced and acidic pH-intensified endothelial dysfunction, but the GPR4 inhibitor did not show any protective effects. These sets of data convince us to propose that activation of GPR68 mediates acidosis alone or synergistically with LPS-induced endothelial injuries. In support of our results, recent studies have identified GPR68 as a mediator of intestinal inflammation [17,41] and cardiac inflammation under chronic kidney disease conditions [34]. Accordingly, inhibition of GPR68 attenuates inflammation and improves the outcomes in murine disease models of colitis and chronic kidney failure-induced cardiac inflammation and fibrosis [16,33].

## 5. Conclusions

In summary, a shift towards an acidic pH appears to be a strong trigger for lung endothelial dysfunction that may have important clinical relevance since acid accumulation is a feature of various disease states such as ischemia. Acidosis also seems to intensify existing inflammatory responses, and GPR68 turns out to be an essential mediator of acidosis-driven lung endothelial injuries. Most importantly, the GPR68-targeting small molecule inhibitor OGM-8345 exerts robust protection against acidosis-induced endothelial dysfunction, suggesting that it could be considered as a potential therapeutic drug candidate to restore endothelial function and treat associated lung injuries.

## Figures and Tables

**Table 1:** List of primer sequences used for RT-PCR.

Gene Name	Forward Primer	Reverse Primer
Human TNF-α	AGGACGAACATCCAACCTTCCCAA	TTTGAGCCAGAAGAGGTTGAGGGT
Human VCAM-1	CAGTAAGGCAGGCTGTAAAAGA	TGGAGCTGGTAGACCCTCG
Human ICAM-1	TTGGGCATAGAGACCCCGTT	GCACATTGCTCAGTTCATACACC
Human IL-6	CCTGAACCTTCCAAAGATGGC	TTCACCAGGCAAGTCTCCTCA
Human IL-8	TGACTTCCAAGCTGGCCGTGG	ACTGCACCTTCACACAGAGCTGC
Human IL-1β	CTCGCCAGTGAAATGATGGCT	GTCGGAGATTCGTAGCTGGAT
Human CXCL5	TGGACGGTGGAAACAAGG	CTTCCCTGGGTTCAGAGA
Human CXCL10	GGAACCTCCAGTCTCAGCACC	GCGTACAGTTCTAGAGAGAGGTAC
Human E-selectin	GAA GGA TGG ACG CTC AAT GG	TGG ACT CAG TGG GAG CTT CAC
Human GAPDH	ATGGGGAAGGTGAAGGTC	GGGGTCATTGATGGCAACAATA
Mouse TNF-α	CTGTAGCCCACGTCGTAGC	TTGAGATCCATGCCGTTG
Mouse VCAM-1	ACGAGGCTGGAATTAGCAGA	TCGGGCACATTTCCACAAG
Mouse ICAM-1	CTGCCTCTGAAGCTCGGATA	GTCACCTCTACCAAGGCAGT
Mouse IL-6	CCGGAGAGGAGACTTCACAG	TCCACGATTTCCCAGAGAAC
Mouse IL-1β	GAAATGCCACCTTTTGACAGTG	TGGATGCTCTCAGGACAG
Mouse KC	GCGCCCAAACCGAAGTCATA	ATGGGGGATGCAGGATTGAG
Mouse CXCL2	CGCTGTCAATGCCTGAAGAC	ACACTCAAGCTCTGGATGTTCTTG
Mouse GAPDH	AATGTGTCCGTCGTGGATCT	AGACAACCTGGTCCTCAGTG

## Data Availability

The original contributions presented in the study are included in the article, further inquiries can be directed to the corresponding author.
